# Chitosan Protects Peripheral Nerves Against Damage Induced by Diabetes Mellitus

**DOI:** 10.3390/life15121860

**Published:** 2025-12-04

**Authors:** Anca-Maria Țucă, Carmen Albu, Alexandra Nicoleta Preda, Alexandra Oltea Dan, Elena-Anca Târtea, Andrei Greșiță, Denisa Floriana Vasilica Pîrșcoveanu, Veronica Sfredel, Smaranda Ioana Mitran, Georgică Târtea

**Affiliations:** 1 Experimental Research Centre for Normal and Pathological Aging, University of Medicine and Pharmacy of Craiova, 2 Petru Rares Street, 200349 Craiova, Romania; anca.tuca@umfcv.ro (A.-M.Ț.); departament2.medicina@umfcv.ro (A.N.P.); alexandra.dan@umfcv.ro (A.O.D.); andrei.gresita@umfcv.ro (A.G.); veronica.sfredel@umfcv.ro (V.S.); smaranda.mitran@umfcv.ro (S.I.M.); georgica.tartea@umfcv.ro (G.T.); 2 Department of Neurology, University of Medicine and Pharmacy of Craiova, 2 Petru Rares St., 200349 Craiova, Romania; carmen.albu@umfcv.ro (C.A.); denisa.pirscoveanu@umfcv.ro (D.F.V.P.); 3 Department of Cardiology, University of Medicine and Pharmacy of Craiova, 2 Petru Rares St., 200349 Craiova, Romania

**Keywords:** chitosan, diabetes mellitus, diabetic peripheral neuropathy

## Abstract

Background: Diabetic peripheral neuropathy (DPN) is one of the most common and debilitating complications of diabetes mellitus, for which current therapies do not prevent nerve degeneration. Chitosan, a biocompatible polysaccharide with antioxidant, anti-inflammatory, and lipid-lowering properties, may exert direct neuroprotective effects. This study evaluated the impact of oral administration of chitosan on peripheral nerve function and structure in a murine model of streptozotocin (STZ)-induced diabetes. Methods: Male C57BL/6 mice were divided into three groups: Sham, untreated diabetics (T1DM) and diabetics treated with chitosan (150 mg/kg/day, 12 weeks). Metabolic, behavioral (Open Field), nociceptive (Von Frey, Tail-Flick), electrophysiological (compound motor action potential—CMAP) and histological (intraepidermal nerve fiber density—IENF) parameters were analyzed. Results: Chitosan did not significantly modify blood glucose (*p* = 0.3366), but showed favorable metabolic effects, reducing LDL cholesterol in T1DM+Chitosan vs. T1DM mice (43.75 ± 5.62 mg/dL vs. 82.75 ± 7.65 mg/dL, *p* < 0.0001) as well as triglycerides (103.5 ± 12.8 mg/dL vs. 175.5 ± 22.8 mg/dL, *p* < 0.0001). In nociceptive tests, chitosan ameliorated thermal hyperalgesia (Tail-Flick: T1DM 1.25 ± 0.19 s vs. T1DM+Chitosan 1.54 ± 0.16 s; *p* = 0.0188) and mechanical allodynia (Von Frey: T1DM 0.16 ± 0.07 g vs. T1DM+Chitosan 0.38 ± 0.15 g, *p* = 0.0103). Electrodiagnostically, chitosan improved CMAP amplitude (T1DM 5.756 ± 0.706 mV vs. T1DM + Chitosan 6.756 ± 0.760 mV, *p* = 0.0409) and reduced CMAP duration (3.161 ± 0.217 ms vs. 2.900 ± 0.080 ms, *p* = 0.0273). Histologically, IENF density significantly increased in the treated group (0.01991 ± 0.00246 vs. 0.01512 ± 0.00253 in T1DM; *p* = 0.0200). Conclusions: Oral administration of chitosan confers functional and structural neuroprotection in STZ-induced diabetic neuropathy despite persistent hyperglycemia.

## 1. Introduction

Diabetes mellitus (T1DM) is a chronic metabolic disease characterized by persistent hyperglycemia, which causes multi-organ damage, including the cardiovascular system, kidneys, retina, and peripheral nervous system [1]. Recent estimates from the World Health Organization indicate that approximately 830 million people are currently living with diabetes worldwide, more than half of whom are not receiving adequate treatment [2]. Data from the 11th edition of the International Diabetes Federation (IDF) Diabetes Atlas show that in 2024, approximately 589 million adults (aged 20–79 years), or 11.1% of the global adult population, had diabetes, with projections suggesting an increase to over 850 million adults by 2050 [2]. Complementing these figures, the Global Burden of Disease 2021 study reported 529 million people living with diabetes in 2021 and a nearly doubling of age-standardized prevalence since 1990, highlighting the rapid expansion of the global burden of diabetes, driven in large part by obesity and an aging population [3,4,5].

Diabetic peripheral neuropathy (DPN) is one of the most common and disabling chronic complications of diabetes. DPN encompasses a spectrum of clinical phenotypes, of which distal symmetric polyneuropathy (DSPN) is the most common and is characterized by length-dependent sensory loss, neuropathic pain, and motor impairment [6,7]. Recent comprehensive reviews highlight that 10–15% of newly diagnosed patients with type 2 diabetes already have DSPN, and the prevalence may exceed 50% in people with diabetes duration of more than 10 years [8,9,10]. In addition, DPN is increasingly recognized in people with prediabetes and impaired glucose regulation, indicating that peripheral nerve dysfunction begins early during dysglycemia [11]. Painful diabetic neuropathy (PDN) affects a substantial proportion of patients; recent epidemiological studies report painful neuropathy in approximately 13–35% of people with diabetes, with wide variability depending on diagnostic criteria and population [12]. Beyond neuropathic pain, DPN is a major contributor to falls, gait instability, foot ulcers, lower limb amputations, and reduced quality of life, and is associated with increased mortality and higher healthcare costs [13,14].

The pathogenesis of DPN is complex and multifactorial. Chronic hyperglycemia, dyslipidemia, and insulin resistance trigger a cascade of metabolic and vascular insults, including increased polyol pathway flux, formation of advanced glycation end products, oxidative and nitrosative stress, mitochondrial dysfunction, endothelial injury, and low-grade inflammation [9]. These processes ultimately disrupt the structure and function of myelinated and unmyelinated fibers, Schwann cells, and the neurovascular unit, leading to impaired nerve conduction, axonal degeneration, and loss of intraepidermal nerve fibers (IENFs), as demonstrated in skin biopsy studies [15,16,17]. Oxidative stress and chronic low-level inflammation are recognized as major determinants of microvascular and nerve degeneration in diabetes, being involved in both nephropathy and neuropathy, and the utility of the CRP/albumin ratio as a marker of diabetic neuropathy confirms the central role of systemic inflammation in peripheral damage, justifying the evaluation of agents with antioxidant and anti-inflammatory potential such as chitosan [18,19]. The current standard management of diabetic neuropathy relies on strict metabolic control and modification of cardiovascular risk factors to slow progression, combined with symptomatic analgesic therapies that target neuropathic pain but do not modify the underlying neurodegenerative process [9]. Thus, there is a critical unmet need for disease-modifying strategies that protect or restore peripheral nerve integrity in the context of diabetes. Chitosan, a linear polysaccharide derived from the deacetylation of chitin, has attracted considerable attention as a biomaterial and bioactive compound due to its biocompatibility, biodegradability, low toxicity, and versatility of chemical modification [20,21,22,23]. In the metabolic field, chitosan and its derivatives have been extensively investigated for their antidiabetic potential. Preclinical and clinical studies summarized in recent reviews indicate that chitosan can attenuate diabetic hyperglycemia by reducing hepatic gluconeogenesis, improving glucose uptake by skeletal muscle, and modulating intestinal carbohydrate digestion and glucose absorption [24,25]. In addition, chitosan favorably influences lipid metabolism, body weight, and adipokine profiles, and exerts antioxidant and anti-inflammatory effects, thereby addressing several pathophysiological components of the metabolic syndrome and type 2 diabetes [24,25,26].

These properties have led to its development as a dietary supplement, drug carrier, and component of multifunctional nanoformulations for metabolic diseases [27].

The intersection of antidiabetic and neuroregenerative properties of chitosan raises the possibility that this polymer may exert direct neuroprotective actions in diabetic neuropathy [28,29]. However, most available studies have examined chitosan in the context of focal nerve injury and local application of biomaterials, rather than systemic administration in models of diffuse metabolic neuropathy [28,29]. Similarly, although antidiabetic studies frequently include streptozotocin (STZ)-induced diabetes models and demonstrate improved glycemic control and β-cell protection following oral or parenteral treatment with chitosan, detailed electrophysiological and neurobehavioral outcomes are rarely evaluated [30].

The present work therefore aims to characterize in detail the functional and structural neuroprotective actions of orally administered chitosan in STZ-induced diabetic neuropathy and to discuss their relevance for the development of disease-modifying therapies in DPN.

## 2. Materials and Methods

### 2.1. Study Design and Ethical Considerations

The study was designed as an in vivo, prospective experiment, using a murine model of chemically induced diabetes mellitus to evaluate the neuromorphological, functional, and behavioral changes associated with diabetic neuropathy, as well as to determine the neuroprotective effects of chitosan. All procedures were in accordance with the European directives on the use of animals in research (Directive 86/609/EEC), with the national legislation in force and with the international standards of good practice. The protocol received the approval of the Ethics Committee of the University of Medicine and Pharmacy of Craiova (no. 36/20.01.2023). The experiments were carried out in a FELASA-accredited animal facility, and histological processing was performed at the Center for Research on Microscopic Morphology and Immunological Studies. According to ARRIVE 2.0 requirements, all procedural steps were rigorously documented, and the personnel involved received specific training [31,32].

### 2.2. Laboratory Animals and Maintenance Conditions

A total of 24 male C57BL/6 mice (8–10 weeks, 18–27 g) were used, maintained under SPF conditions, with a 12/12 h light–dark cycle, ambient temperature of 22 ± 1 °C, and humidity of 50–60%. Standard food and water were provided ad libitum, and food consumption was recorded through metabolic cages. To reduce behavioral variability, the animals were handled daily for at least three days before the start of the experiments.

### 2.3. Induction of Diabetes Mellitus and Experimental Allocation

Diabetes was induced by intraperitoneal administration of a single dose of streptozotocin (STZ, 150 mg/kg), prepared extemporaneously in citrate buffer. Blood glucose was monitored weekly by sampling from the caudal vein (Contour Plus One glucometer), and animals were considered diabetic for values ≥ 250 mg/dL.

Then, 72 h after STZ injection, animals were randomized into three groups (*n* = 8/group):

- Sham (DM−): administration of 0.9% NaCl, without STZ; physiological control group.
- Type 1 diabetes mellitus—T1DM (DM+): diabetes induced, without therapeutic intervention.
- T1DM+Chitosan (T1DM+Chitosan): oral administration of chitosan (150 mg/kg/day) by gastric gavage, for 12 weeks.

The chitosan used in our study had a medium molecular weight and was purchased from Sigma-Aldrich (Catalog No. 448877, Sigma-Aldrich Chemie GmbH, Taufkirchen, Germany). The dose employed in the present study has also been used in previous investigations [23,24]. Moreover, several studies have demonstrated that chitosan is absorbed at the level of the intestinal mucosa, and in vivo experiments have shown that its absorption is inversely proportional to its molecular weight [25,26].

During the protocol, body weight, blood glucose, and food and water consumption were monitored. Finally, total cholesterol, LDL cholesterol, HDL cholesterol, and triglycerides were determined in venous blood (collected by puncture of the inferior vena cava after euthanasia of the animal) using the MulticareIN analyzer( Biochemical Systems International, Milan, Italy).

### 2.4. Behavioral Assessments

Behavioral testing was performed at baseline (pre-STZ), once every 3 weeks until week 12.

#### 2.4.1. Open Field Test

Spontaneous locomotor activity and anxiety-related behaviors were assessed using the Open Field Test. Testing was performed in a rectangular transparent Plexiglas arena (50 × 33 × 15 cm), uniformly illuminated and placed in a quiet room to minimize external influences. Each animal was placed individually in the center of the arena at the beginning of the session and was monitored for 10 min, with behavior being video recorded by a top-mounted, fixed-angle camera to ensure capture of the entire testing space. The analysis of movement traces and behavioral parameters was performed using EthoVision XT 14 software (Noldus Information Technology, Wageningen, The Netherlands). Quantified parameters included total distance traveled, average movement speed, time spent in the central area compared to the peripheral area of the arena (automatically defined in the software), number and duration of episodes of continuous locomotion, as well as episodes of immobility. The central area was used as an indicator of anxious behavior, while locomotor variables allowed the assessment of general mobility. To prevent olfactory influences on behavior, the arena was rigorously cleaned with 70% ethanol solution and allowed to dry completely between consecutive testing of the animals.

#### 2.4.2. Mechanical Allodynia Test

Mechanical sensitivity was assessed using calibrated Von Frey filaments, applied to the plantar surface of the left hind paw. Animals were placed individually in test chambers equipped with a metal grid with fine perforations, which allowed access to the tested area without additional constraints. Before measurements, animals were allowed to acclimate to the test environment to reduce the influence of stress or increased motor activity on behavioral responses. The filaments were applied perpendicularly to the plantar surface, with a gradual increase in stimulus intensity, according to the standardized force range of the Von Frey set used. For each specimen, the mechanical threshold was established based on a series of three independent determinations, separated by intervals of at least 10 min, to avoid the phenomenon of desensitization or repetitive learning of the stimulus. Any clear reflex withdrawal behavior, paw shaking, or protective behavior immediately after filament application was considered a positive response. The final mechanical threshold was calculated as the lowest force that generated a consistent positive response in replicates. Testing was performed in a controlled environment, characterized by low lighting, minimal noise, and stable ambient temperature, to limit stress factors that may influence nociceptive processing. All measurements were performed by trained personnel familiar with species-specific behaviors and the interpretation of nociceptive responses.

#### 2.4.3. Tail-Flick Tail Immersion Test

The Tail-Flick Test was used to assess the thermal nociceptive threshold. A thermostatic tank was used for measurements, capable of maintaining the water temperature at 50 ± 0.5 °C, a value chosen to produce a reproducible nociceptive stimulation without inducing tissue damage when applied within the accepted time limits. Before testing, the animals were gently handled to reduce stress and were acclimated to the experimental environment, thus ensuring a stable behavioral response. The assessment consisted of inserting the distal segment of the tail into a standardized point, representing approximately the terminal third, which allows for high comparability between individuals. The nociceptive response was recorded as the latency time to reflex tail withdrawal, using a digital stopwatch to minimize subjective variations. Four determinations were performed for each animal, separated by 5 min intervals, the period necessary for the tissue to recover to its basal state after exposure to the thermal stimulus. The arithmetic mean of these values was used as the final score, thus reducing experimenter-dependent variability. A maximum exposure time of 30 s was established as an ethical limit to prevent any risk of tissue damage, and in the absence of a reflex response, the test was stopped immediately upon reaching this limit. The procedures were performed in accordance with the ethical principles for the use of animals in research, including minimizing pain and discomfort and using the minimum number of subjects necessary to obtain adequate statistical validity.

### 2.5. Tissue Sampling and Histological Analysis

After induction of deep anesthesia, the animals were euthanized according to ethical protocols. For assessment of intraepidermal nerve fibers density (IEFN) we excised bilateral plantar skin tissue and then fixed it in 4% formalin and processed according to standard histological steps: washing for 24 h, embedding in paraffin, sectioning (4 μm) with an HM355S microtome, mounting on poly-L-lysine-treated slides, and incubation at 60 °C for 24 h. Routine staining included hematoxylin–eosin. For IEFN analysis, immunohistochemistry with the PGP 9.5 antibody (Abcam, Recombinant Anti-PGP9.5 antibody [EPR4118]—Neuronal Marker—ab108986, dilution 1:200) was used to assess neuromorphological integrity, with DAB development and counterstaining with Mayer hematoxylin. Slides were examined under a Nikon 55i microscope (Apidrag, Bucharest, Romania) and subsequently digitized with a MoticEasyScan Pro 6 at 20× (Kowloon, Hong Kong). Quantitative analyses (relative fibrous nervous tissue area—expressed as μm^2^/μm^2^) were performed with Image ProPlus AMS 9 (Media Cybernetics, Rockville, MD, USA). Initially we measured the entire cross-sectional area; then we measured the area of the nerve fibers and related it to the total cross-sectional area. Histological evaluation was performed by two independent observers, blinded to experimental allocation.

### 2.6. Electrophysiological Evaluations

To evaluate the electrophysiological properties, the compound muscle action potential (CMAP) was recorded, for which both duration and amplitude were analyzed. Electrical stimulation of a motor nerve generates an impulse that propagates along its fibers to the innervated muscle. The resulting contraction produces an electrical activity that reflects the recruitment of all muscle fibers served by that nerve; this activity is expressed by CMAP. The CMAP amplitude, measured in millivolts (mV), indicates the number of activated muscle fibers: low values suggest axonal loss (decrease in the number of functional nerve fibers), while a prolonged duration indicates a reduction in conduction velocity, characteristic of demyelination processes. In mice, the CMAP of the sciatic nerve is recorded at the level of the musculature it innervates; in the present study, measurements were performed at the level of the gastrocnemius muscle. Electrophysiological monitoring was performed with a Neuro MEB-4 electromyography system (Neurosoft Ltd., Ivanovo, Russia), equipped with dedicated software, version 4.0. Throughout the experiments, the depth of anesthesia—induced by a mixture of ketamine and xylazine—was checked by the pinch reflex, to confirm the absence of nociceptive responses. To protect the cornea, Bepanthen ophthalmic ointment (Bayer, Leverkusen, Germany) was applied. Also, the limb temperature was maintained within physiological limits throughout the procedure (maintained at 36–37 °C). Electroneurographic recordings were performed with four monopolar needle-type electrodes (SEI EMG, Cittadella, Italy): two used for nerve stimulation and two for recording electrical responses at the level of the examined nerve. The stimulating electrodes were inserted subcutaneously along the course of the sciatic nerve, at the proximal portion of the thigh, ensuring that the electrical impulse was delivered directly to the main nerve trunk. The recording electrodes were positioned distally, in the muscle territory innervated by the sciatic nerve. Specifically, one electrode was placed in the belly of the gastrocnemius muscle to capture the CMAP, while the reference electrode was inserted adjacent to it, in the distal region of the hind limb. Acquisition parameters were frequency band 20 Hz–10 kHz, sweep speed 1 ms/division and sensitivity 5 mV/division. Each animal was administered three electrical stimuli, each with a duration of 0.2 ms. The intensity was 5 mA for the first stimulus, and for the next two it was increased to 6 mA if the CMAP amplitude did not increase significantly. In two isolated situations, to obtain the maximum motor response, the intensity was adjusted to 7 mA. For each subject, the final values analyzed represented the average of the highest CMAP amplitudes and durations obtained. Before the induction of experimental diabetes by STZ administration, all animals underwent preliminary electroneurographic recordings to establish reference values.

### 2.7. Statistical Analysis

Data were expressed as mean ± standard deviation. Statistical analysis was performed in GraphPad Prism 9, after preliminary collection in Microsoft Excel. Normality of distribution was tested before the selection of parametric tests. Inter-group differences were evaluated by ANOVA followed by appropriate post hoc tests. The significance threshold was set at *p* < 0.05; significance levels were noted: * (*p* < 0.05), ** (*p* < 0.01), *** (*p* < 0.001).

## 3. Results

### 3.1. Assessment of the Impact of Streptozotocin Administration

#### 3.1.1. Plasma Glucose Level

After the induction of diabetes with STZ, mice in the T1DM and T1DM+Chitosan groups showed a marked increase in blood glucose at week 9, reaching values of approximately 450–470 mg/dL, compared to Sham animals, which were maintained at physiological levels of ~110–120 mg/dL (e.g., 112.75 ± 24.5 mg/dL). 2-way ANOVA analysis indicates significant differences between the Sham group and the two diabetic groups at each time point analyzed (e.g., at 6–7 weeks after the onset of diabetes mellitus: Sham vs. T1DM: mean diff = −328.4 mg/dL, *p* < 0.0001; Sham vs. Chitosan: mean diff = −350.6 mg/dL, *p* < 0.0001).

Oral administration of chitosan did not produce a statistically significant reduction in blood glucose compared to untreated diabetic mice (e.g., week 6 after diabetes onset: T1DM vs. T1DM+Chitosan: mean diff = −22.25 mg/dL, *p* = 0.3366), although the values tended to be slightly lower at some time points. Therefore, the beneficial effects observed on later functional and behavioral parameters are not due to a glycemic normalization, suggesting a mechanism independent of the decrease in blood glucose.

#### 3.1.2. Body Weight

Sham animals maintained a stable weight in the range of 25–27 g throughout the experiment (e.g., 26.31 ± 0.89 g at 11 weeks). In contrast, untreated diabetic mice showed a progressive weight reduction, reaching ~21 g at the end (21.241 ± 1.64 g). Both T1DM and T1DM+Chitosan groups showed significant weight decreases compared to Sham at all time points analyzed (e.g., at the end of the experiment: Sham vs. T1DM: mean diff = 4.724 g, *p* = 0.0003; Sham vs. Chitosan: mean diff = 5.160 g, *p* < 0.0001). Chitosan did not significantly change weight compared to the untreated diabetic group (e.g., at the end of the experiment: T1DM vs. T1DM+Chitosan, *p* = 0.8801). This suggests that the treatment does not influence the catabolic status caused by diabetes but does not worsen weight loss either.

#### 3.1.3. Water Intake

As is characteristic of STZ-induced diabetes, T1DM mice developed severe polydipsia, reaching mean values of ~40–45 mL/day, while Sham animals remained at 4–6 mL/day. Statistical analysis confirms major differences between the Sham and diabetic groups at all post-STZ time points (e.g., at the end of the experiment: Sham vs. T1DM: mean diff = −36.30 mL, *p* < 0.0001). Chitosan did not significantly alter water intake compared to T1DM (e.g., T1DM vs. Chitosan: mean diff = −1.625 mL, *p* = 0.7945). Therefore, the treatment did not influence polydipsia associated with hyperglycemia, which is consistent with the lack of glycemic normalization.

#### 3.1.4. Food Intake

Food consumption increased markedly in the untreated diabetic group, reaching values of 5–6 g/day at some times, compared to the Sham group, which remained between 3.8 and 4.2 g/day. Statistical analyses show consistent differences between the Sham and diabetic groups (e.g., at 9–10 weeks after the onset of diabetes mellitus: Sham vs. T1DM: mean diff = −1.250 g, *p* = 0.0017). Chitosan induced a slight reduction in food intake compared to the T1DM group, but the effect is not statistically significant (e.g., at 9–10 weeks after the onset of diabetes mellitus: T1DM vs. Chitosan: *p* = 0.2882). Although the trend is downward, the magnitude of the effect is limited.

**Figure 1:**
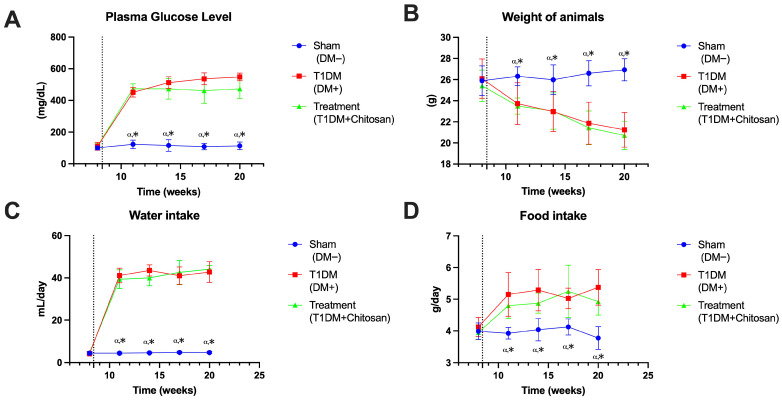
Evolution of metabolic parameters in Sham, untreated diabetic (T1DM), and diabetic mice treated with chitosan (T1DM+Chitosan). (**A**) Plasma glucose level (mg/dL). (**B**) Body weight (g). (**C**) Water consumption (mL/day). (**D**) Food consumption (g/day). Data are expressed as mean ± SD. Statistical significance: ^α^ *p* < 0.05 Sham vs. T1DM, * *p* < 0.05 Sham vs. T1DM+Chitosan. The dotted line represents streptozotocin administration.

Figure 1 demonstrates the establishment of a robust diabetogenic metabolic phenotype in the T1DMand T1DM+Chitosan groups, characterized by severe and persistent hyperglycemia, progressive weight loss, polydipsia, and hyperphagia. Oral administration of chitosan did not significantly modify major metabolic parameters (glycemia, water consumption, food consumption, body weight) compared to untreated diabetic mice. This observation supports the hypothesis that the neuroprotective effects presented later in the article are at least partially independent of glycemic control, representing a direct action on the peripheral nerve.

### 3.2. Effects on Lipid Profile

**Figure 2:**
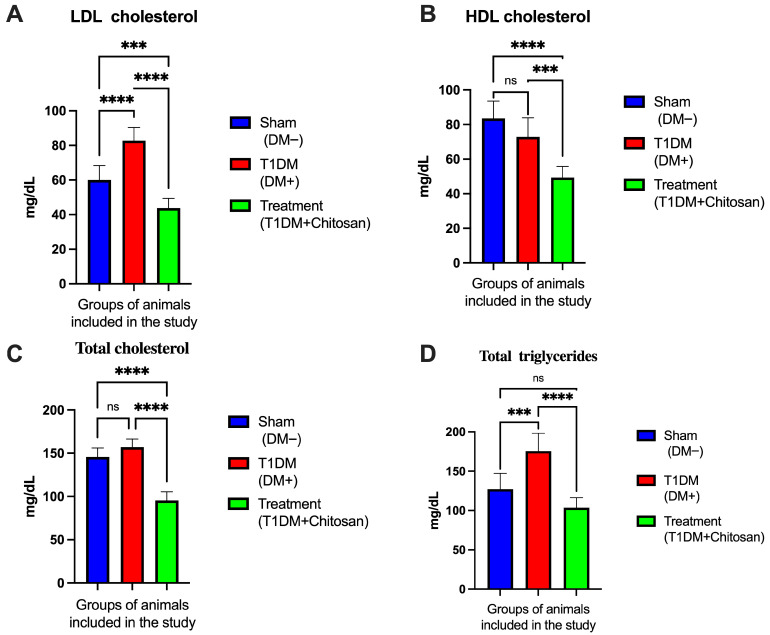
Serum lipid profile in Sham mice, untreated diabetic mice (T1DM), and diabetic mice treated with chitosan (T1DM+Chitosan). (**A**) LDL cholesterol. (**B**) HDL cholesterol. (**C**) Total cholesterol. (**D**) Total triglycerides. Data are presented as mean ± SD. Statistical significance: *** *p* < 0.001; **** *p* < 0.0001. ns: not significant.

The effects of STZ-induced diabetes and chitosan treatment on lipid profile, including LDL cholesterol, HDL cholesterol, total cholesterol, and triglycerides are illustrated in Figure 2. One-way ANOVA followed by Tukey post hoc test revealed significant differences between the three experimental groups.

#### 3.2.1. LDL Cholesterol

Untreated diabetic mice (T1DM) showed significantly increased LDL cholesterol values (82.75 ± 7.65 mg/dL) compared to Sham animals (60.00 ± 8.36 mg/dL). The difference was statistically significant (mean diff. = −22.75 mg/dL, *p* < 0.0001). Chitosan administration resulted in a substantial reduction in LDL cholesterol, the mean in the T1DM + Chitosan group being 43.75 ± 5.62 mg/dL, significantly lower than both the T1DM + group (mean diff. = 39.00 mg/dL, *p* < 0.0001) and Sham (mean diff. = 16.25 mg/dL, *p* = 0.0006). These results suggest a marked lipid-lowering effect of chitosan in diabetic mice.

#### 3.2.2. HDL Cholesterol

Regarding HDL cholesterol, Sham animals presented the highest values (83.50 ± 10.0 mg/dL). Induction of diabetes moderately reduced HDL (72.88 ± 11.0 mg/dL), but the difference Sham vs. T1DM did not reach statistical significance (mean diff. = 10.63 mg/dL, *p* = 0.0824). Chitosan treatment significantly decreased HDL cholesterol compared to both control groups, with a mean of 49.38 ± 6.43 mg/dL. The differences T1DM + Chitosan vs. Sham (mean diff. = 34.13 mg/dL, *p* < 0.0001) and T1DM + Chitosan vs. DM+ (mean diff. = 23.50 mg/dL, *p* = 0.0002) were statistically significant. Although the decrease in HDL is not metabolically favorable, it must be interpreted in the general context of the lipid profile and potential mechanisms specific to chitosan.

#### 3.2.3. Total Cholesterol

Total cholesterol followed a similar evolution. The Sham group showed values of 145.8 ± 10.28 mg/dL, comparable to those in the T1DM + group (157.1 ± 9.37 mg/dL, *p* = 0.0787, not significant).

In contrast, the chitosan-treated group showed a marked reduction in total cholesterol (95.38 ± 10.07 mg/dL). The differences were significant both compared to the Sham group (mean diff. = 50.38 mg/dL, *p* < 0.0001) and to T1DM + (mean diff. = 61.75 mg/dL, *p* < 0.0001). This result confirms the robust hypocholesterolemic action of chitosan.

#### 3.2.4. Triglycerides

Diabetes induction significantly increased triglyceride levels (175.5 ± 22.8 mg/dL) compared to the Sham group (127.1 ± 20.0 mg/dL, mean diff. = −48.38 mg/dL, *p* = 0.0001). Chitosan treatment reduced triglycerides to 103.5 ± 12.81 mg/dL, a level comparable to that of the Sham group (mean diff. Sham vs. Chitosan = 23.63 mg/dL, *p* = 0.0542, non-significant). However, the difference compared to the T1DM + group was significant (mean diff. = 72.00 mg/dL, *p* < 0.0001). Therefore, chitosan almost completely reversed diabetes-induced hypertriglyceridemia.

Overall, Figure 2 highlights important metabolic effects of chitosan on the lipid profile of diabetic mice. Although HDL cholesterol decreased below the levels of the other groups, the significant reduction in LDL cholesterol, triglycerides, and total cholesterol suggests a substantial overall lipid-lowering effect. This metabolic profile is favorable in the context of diabetes and may contribute to the amelioration of inflammatory and oxidative processes involved in diabetic neuropathy.

### 3.3. Electroneurography

**Figure 3:**
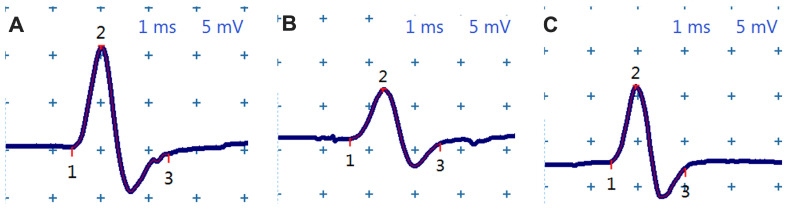
Representative traces of compound muscle potential (CMAP) recorded in the gastrocnemius muscle. (**A**) Sham mice without diabetes mellitus. (**B**) Untreated diabetic mice (T1DM). (**C**) Diabetic mice treated with chitosan (T1DM + Chitosan). 1 is the onset CMAP, 2 is the peak of CMAP, 3 is the end of CMAP.

The electrophysiological evaluation of the sciatic nerve revealed clear changes induced by experimental diabetes and a partial ameliorating effect of chitosan treatment. Analysis of the CMAP (Figure 3) showed that mice in the Sham group presented compound motor potentials with normal morphology, characterized by high amplitude and short duration, reflecting the axonal and myelin integrity of the peripheral nerve. In contrast, untreated diabetic animals presented an obvious reduction in amplitude, a broadening of the potential and a visible prolongation of the duration, aspects consistent with the installation of an axonal and demyelinating diabetic neuropathy. In the diabetic group treated with chitosan, the CMAP shape partially approached the normal morphology, with higher potentials and shorter durations than in the T1DM group, suggesting a neuroprotective effect of the treatment.

**Figure 4:**
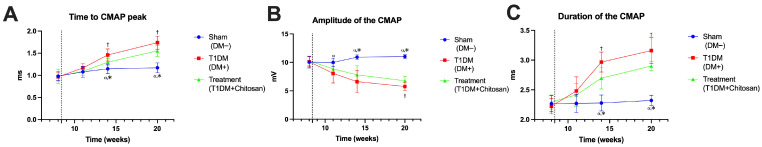
Electrophysiological parameters of CMAP in evolution in Sham, diabetic (T1DM), and diabetic mice treated with chitosan (T1DM + Chitosan). (**A**) Time to peak. (**B**) CMAP amplitude. (**C**) CMAP duration. ^α^ *p* < 0.05 vs. T1DM, * *p* < 0.05 vs. T1DM+Chitosan, † *p* < 0.05 vs. T1DM+Chitosan. The dotted line represents streptozotocin administration.

Quantitative analysis of electrophysiological parameters (Figure 4) confirms these observations. The time to peak CMAP remained stable in the Sham group throughout the experiment, ranging between 0.98 and 1.16 ms. In diabetic mice, however, this latency increased progressively, reaching 1.738 ± 0.146 ms at 20 weeks, significantly higher than in the control group (1.169 ± 0.111 ms, *p* < 0.0001), indicating a marked slowing of nerve conduction. Chitosan administration moderated this alteration, with values in the T1DM + Chitosan group being significantly lower than in the untreated diabetic group (1.553 ± 0.125 ms, *p* = 0.0418), although without completely returning to physiological levels. Regarding the CMAP amplitude, considered the main electrophysiological marker of axonal integrity, a marked reduction was observed in the diabetic group. At 20 weeks, the mean amplitude decreased from 11.025 ± 0.328 mV in the Sham group to 5.756 ± 0.706 mV in the T1DM group, a highly statistically significant difference (*p* < 0.0001). Chitosan treatment caused a partial recovery of the amplitude, which increased to 6.756 ± 0.760 mV, this value being significantly higher than in the untreated diabetic group (*p* = 0.0409), suggesting partial axonal protection. The CMAP duration, a parameter sensitive to the processes of demyelination and conduction dispersion, followed the same pattern. In the Sham group, this remained constant around normal values (~2.26–2.32 ms), but in the diabetic group it gradually increased, finally reaching 3.161 ± 0.217 ms, significantly higher than in the control group (*p* < 0.0001). Chitosan treatment significantly reduced this prolongation, with the CMAP duration being 2.900 ± 0.080 ms, statistically different from that of the T1DM group (*p* = 0.0273), indicating a decrease in myelin damage. Electrophysiological data show that STZ-induced diabetes produces a consistent degradation of peripheral nerve function, manifested by a decrease in CMAP amplitude, a prolongation of the latency to peak, and an increase in the duration of the potential. Oral administration of chitosan significantly attenuates all these changes, suggesting that its neuroprotective effects are exerted at both axonal and myelin levels, even in the absence of a marked reduction in blood glucose.

### 3.4. Behavioral Assessment

#### 3.4.1. Open Field Test

**Figure 5:**
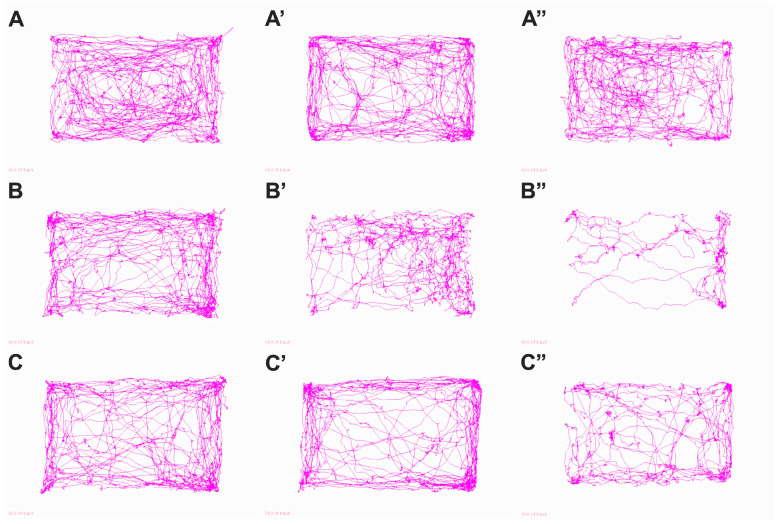
Representations of locomotor trajectories in the Open Field Test at the beginning, middle (′) and end of the experiment (″). (**A**–**A″**) Sham mice without diabetes mellitus. (**B**–**B″**) Untreated diabetic mice (T1DM). (**C**–**C″**) T1DM mice treated with chitosan.

**Figure 6:**
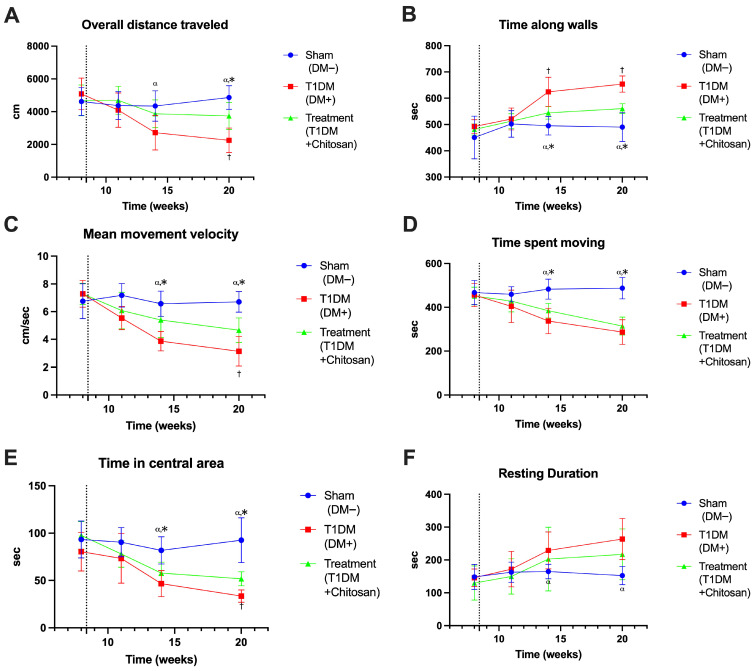
Quantitative behavioral analysis in the Open Field Test. (**A**) Total distance traveled. (**B**) Time spent along the walls. (**C**) Average movement speed. (**D**) Time spent moving. (**E**) Time spent in the central area. (**F**) Duration of rest periods. Data are expressed as mean ± SD. ^α^ *p* < 0.05 vs. T1DM, * *p* < 0.05 vs. T1DM+Chitosan, ^†^ *p* < 0.05 vs. T1DM+Chitosan. The dotted line represents streptozotocin administration.

Behavioral analysis in the Open Field (OF) Test revealed progressive changes in locomotor activity, anxiety, and exploratory motivation induced by experimental diabetes, as well as a partial ameliorating effect of chitosan treatment. The graphical representations of the movement trajectories (Figure 5) and the quantification of locomotor parameters (Figure 6) converge towards the same conclusion: diabetes strongly compromises mobility and reduces exploratory activity, and chitosan significantly attenuates these effects.

Qualitatively, the trajectories exemplified in Figure 5 demonstrate that mice in the Sham group (A,A′,A″) exhibit an activity uniformly distributed throughout the arena, with ample movements and active exploration of the central area. In contrast, untreated diabetic animals (B,B′,B″) show a pronounced restriction of the paths, with the predominance of movements along the walls, the reduction in the explored area, and an evident fragmentation of the paths, an aspect that becomes more pronounced towards the end of the experiment. Animals treated with chitosan (C,C′,C″) show more extended and more homogeneous paths compared to the untreated diabetic group, suggesting an improvement in the exploratory capacity and an attenuation of the anxious behavior. These visual observations are supported by the parametric analysis presented in Figure 6.

The total distance traveled was significantly reduced in diabetic mice. While Sham animals maintained values of approximately 4615 ± 847 cm at 8 weeks and 4863 ± 722 cm at the end, the T1DM group showed a progressive reduction, reaching only 2252 ± 746 cm at the end. The difference between Sham and T1DM was significant as early as week 14 (mean diff. = 1628 cm, *p* = 0.0144) and was accentuated at 20 weeks (mean diff. = 2611 cm, *p* < 0.0001). Chitosan increased the distance traveled compared to T1DM (3736 ± 819 cm, *p* = 0.0054), indicating a clear improvement in spontaneous mobility. Regarding the time spent along the walls, a parameter associated with anxiety, diabetic mice showed significantly increased values. At 20 weeks, T1DM reached 653.9 ± 30.9 s, compared to 490.3 ± 55.1 s in the Sham group (*p* < 0.0001). Chitosan significantly reduced this avoidance behavior, with the time spent at the edge decreasing to 560.4 ± 19.0 s (*p* = 0.0108 vs. T1DM), suggesting a decrease in anxiety levels. The same trend was observed for the time spent in the central area, which was drastically reduced in diabetic animals. At 20 weeks, Sham mice spent 92.5 ± 23.6 s in the center, while T1DM only 33.6 ± 6.6 s (mean diff. = 58.9 s, *p* = 0.0003). Chitosan ameliorated this decrease, with treated animals spending 51.8 ± 7.4 s, a significant increase over T1DM (*p* = 0.0004), suggesting a reduction in anxiety behavior and an improvement in motivation to explore. The average speed of movement followed the same pattern. The Sham group maintained values of 6.7–7.7 cm/s, while T1DM decreased to 3.13 ± 1.06 cm/s at 20 weeks (*p* < 0.0001 vs. Sham). Chitosan significantly increased the speed compared to the diabetic group (mean diff. = −1.53 cm/s; *p* = 0.0194), with the final values being 4.66 ± 0.88 cm/s, reflecting an improvement in locomotor performance. Another relevant indicator, the time spent moving, decreased significantly in diabetic mice, from 487 ± 49 s in Sham, to only 286 ± 56 s in T1DM (*p* < 0.0001). Chitosan improved this parameter, with treated animals recording 314 ± 41 s, significantly different from T1DM (*p* < 0.0001), although it did not return to physiological levels. In addition, resting time was significantly increased in diabetic mice, reaching 263.8 ± 62.7 s, compared to 152.3 ± 27.8 s in the Sham group (*p* = 0.0028). Chitosan reduced the tendency towards inactivity, with the treated group showing 217.3 ± 77.3 s, although the difference compared to T1DM did not reach the threshold of significance at all experimental times.

Behavioral data show that STZ-induced diabetes significantly reduces mobility, speed, and time spent in exploratory areas and increases anxiety levels, reflected by avoidance of the central area and preference for the edges. Chitosan treatment consistently attenuates these deficits, improving mobility, decreasing anxiety, and partially restoring normal exploratory behaviors.

#### 3.4.2. Tail-Flick Tail Immersion Test

Evaluation of nociceptive sensitivity by Tail-Flick and mechanical allodynia tests showed that STZ-induced diabetes produces a progressive deterioration of peripheral sensory function, characterized by reduced latency to thermal stimulus and decreased mechanical threshold, changes that are partially ameliorated following chitosan administration. In the Tail-Flick Test, Sham mice maintained stable tail withdrawal latency values throughout the experiment, ranging between 2.51 ± 0.38 s (11 weeks) and 2.75 ± 0.22 s (20 weeks). In contrast, untreated diabetic animals showed a steep decrease in latency as early as week 11, dropping to 1.71 ± 0.12 s, and at the end of the experimental period reaching only 1.25 ± 0.19 s, a value significantly lower than that of the Sham group (mean difference = 1.49 s, *p* < 0.0001). This result indicates the installation of thermal hypersensitivity, a phenomenon characteristic of diabetic sensory neuropathy. Chitosan treatment visibly attenuated this decrease. Although T1DM+Chitosan animals did not reach the physiological level of the Sham group, their latency remained significantly higher than in the untreated diabetic group throughout the monitoring. At 20 weeks, the values were 1.54 ± 0.16 s, the difference from T1DM being statistically significant (mean difference = 0.28 s, *p* = 0.0188). This effect suggests that oral administration of chitosan reduces the thermal hyperalgesia characteristic of diabetic neuropathy.

#### 3.4.3. Mechanical Allodynia Test

**Figure 7:**
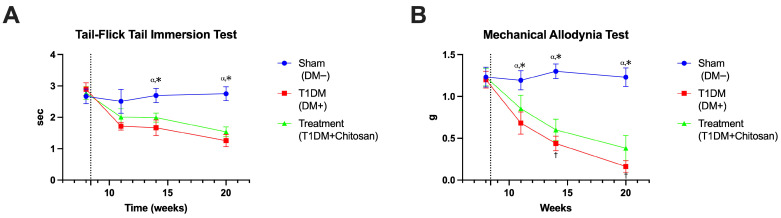
Evaluation of thermal and mechanical sensitivity in Sham (DM−), untreated diabetic (T1DM) and diabetic (T1DM+Chitosan) mice. (**A**) Tail-Flick Tail Immersion Test. (**B**) Mechanical Allodynia Test. Data are expressed as mean ± SD. ^α^ *p* < 0.05 vs. T1DM, * *p* < 0.05 vs. T1DM+Chitosan, † *p* < 0.05 vs. T1DM+Chitosan. The dotted line represents streptozotocin administration.

Concordant results were also obtained in the mechanical allodynia test (Von Frey). In the Sham group, the withdrawal threshold to mechanical stimulus remained constant in the range of 1.19–1.30 g, reflecting intact sensory function. In contrast, in the untreated diabetic group, the threshold underwent a drastic decrease: from 1.20 ± 0.10 g (8 weeks) to only 0.16 ± 0.07 g at the end of the experiment. This strong reduction, statistically significant compared to Sham (mean difference = 1.07 g, *p* < 0.0001), indicates the installation of a severe mechanical allodynia, a consequence of the damage to small and medium sensory fibers. Chitosan administration significantly reduced the severity of mechanical allodynia. At 20 weeks, the withdrawal threshold in the treated group was 0.38 ± 0.15 g, significantly higher than in untreated diabetic mice (mean difference = 0.22 g, *p* = 0.0103), but still below the physiological level. The results confirm that the treatment does not completely normalize sensitivity but slows down the progressive deterioration and partially preserves sensory function. Thus, Figure 7 demonstrates that STZ induced diabetes causes a marked decrease in the latency of response to thermal stimuli and a drastic reduction in mechanical thresholds, reflecting the accelerated degeneration of peripheral sensory afferents. Chitosan administration significantly attenuates both thermal hyperalgesia and mechanical allodynia, supporting the hypothesis of its direct neuroprotective effects on sensory fibers, independent of strict glycemic control. These data are consistent with the electrophysiological and histological findings described previously, reinforcing the role of chitosan as a potential modifying agent in diabetic neuropathy.

### 3.5. Histological Evaluation

**Figure 8:**
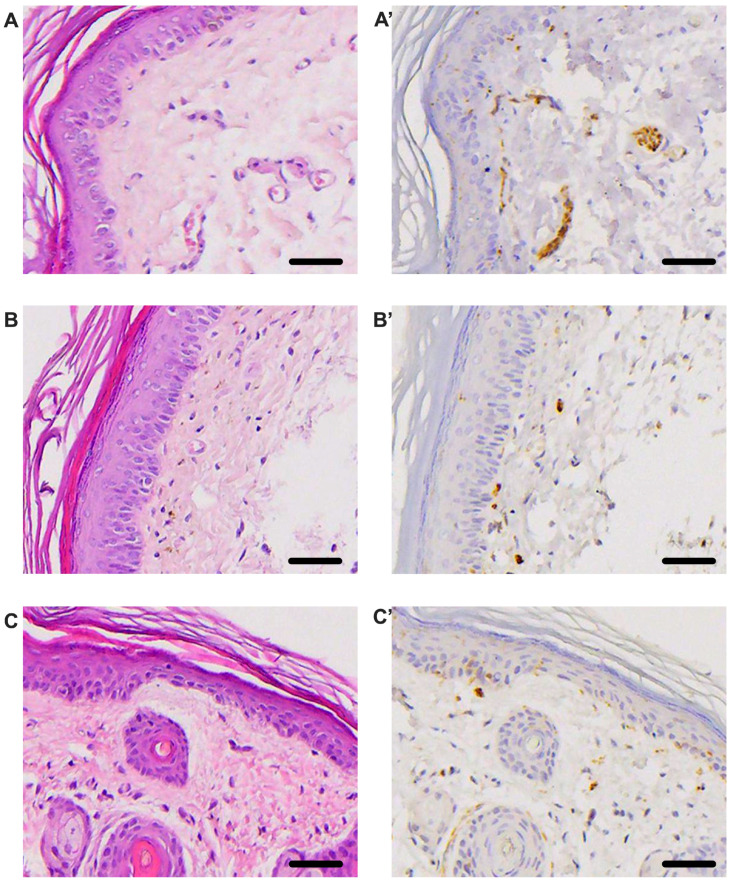
Plantar skin histology in Sham mice (DM−, (**A**,**A′**)), untreated diabetics (T1DM, (**B**,**B′**)) and diabetics treated with chitosan (T1DM+Chitosan, (**C**,**C′**)). Hematoxylin and eosin images (**A**–**C**) and PGP 9.5 immunohistochemistry (**A′**–**C′**). Scale bar: 10 μm.

Histological analysis of plantar skin (Figure 8) revealed clear structural changes associated with streptozotocin-induced diabetes, both in the epidermis and dermis, and in the distribution of small sensory nerve fibers. In the Sham group (A and A′), HE staining showed a normal epidermis, with a uniform stratum corneum and a well-organized dermal architecture. Immunostaining with PGP 9.5 (A′) revealed the presence of numerous intraepidermal nerve fibers, in the form of well-defined, filiform profiles, which penetrate the basal and suprabasal layers of the epidermis in an orderly manner. This increased density of small sensory fibers is typical of neurophysiologically intact skin tissue. In contrast, in untreated diabetic animals (B and B′), HE staining revealed a discrete thinning of the epidermis, focal degeneration of the spinous layer, and a reduction in the consistency of the dermal matrix, suggesting altered microvascularization and tissue homeostasis. PGP 9.5 immunostaining (B′) showed a marked decrease in intraepidermal nerve fibers, with the presence of only a few short, dispersed fragments, and an almost complete absence of the fine terminal arborizations characteristic of a healthy skin. This visible decrease corresponds to a severe axonal loss, consistent with small-fiber diabetic sensory neuropathy. Chitosan treatment (C and C′) produced a clear improvement in these changes. Histologically, the epidermis retains its thickness and stratification, and the dermis presents a more compact structure than in the untreated diabetic group. PGP 9.5 staining (C′) reveals a higher density of intraepidermal nerve fibers compared to the T1DM group, with the presence of preserved filiform nerve arborizations, although not to the level observed in normoglycemic skin. This effect suggests the ability of chitosan to protect and preserve small sensory nerve fibers, consistent with the functional improvements observed in nociception tests.

**Figure 9:**
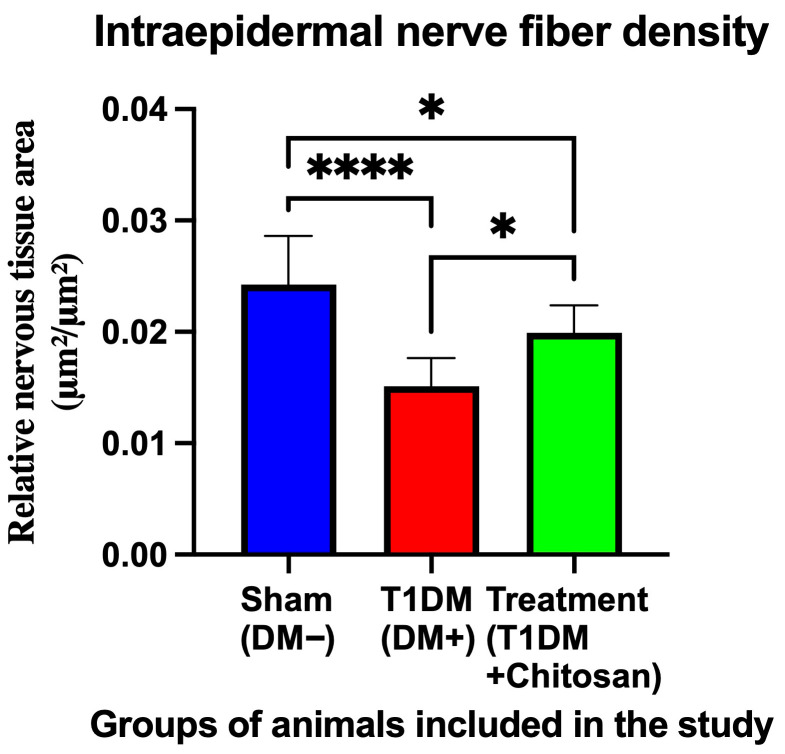
Intraepidermal nerve fiber (IENF) density in plantar skin. Data are expressed as mean ± SD. Statistical significance: * *p* < 0.05; **** *p* < 0.0001.

Quantitative analysis (Figure 9) confirms the visual differences. The relative density of intraepidermal nerve fibers (expressed as μm^2^/μm^2^) was 0.02422 ± 0.00438 in the Sham group. In untreated diabetic mice, the density decreased to 0.01512 ± 0.00253, representing a significant reduction (mean difference = 0.00910, *p* < 0.0001). Chitosan treatment significantly increased IENF density to 0.01991 ± 0.00246, intermediate between Sham and T1DM, but significantly higher than in the untreated diabetic group (mean difference = 0.00431, *p* = 0.0200). The difference compared to the Sham group remains significant (*p* = 0.0379), suggesting that although chitosan does not completely restore normal sensory architecture, it clearly reduces the severity of diabetes-induced axonal loss.

These results demonstrate that diabetes causes a profound loss of small intraepidermal sensory fibers, the integrity of which is essential for the transmission of nociceptive and tactile-protective information. Oral administration of chitosan attenuates this degeneration, preserving a significant proportion of fibers, which is in full agreement with the effects observed in behavioral tests (mechanical allodynia and thermal hyperalgesia) and in electrophysiological assessments of the sciatic nerve. Overall, the data suggest a direct neuroprotective effect of chitosan on small peripheral nerve fibers, a fundamental component in the pathogenesis of diabetic neuropathy.

## 4. Discussion

The results of the present study show that oral administration of chitosan, in a fixed dose and over the long term, confers significant functional and structural protection to the peripheral nerve in a murine model of STZ-induced diabetic neuropathy, in the absence of a notable improvement in hyperglycemia. Chitosan improved sciatic nerve electrophysiological parameters (CMAP amplitude, latency to peak, potential duration), reduced thermal hyperalgesia and mechanical allodynia, ameliorated locomotor and anxiety deficits in the Open Field, and preserved intraepidermal nerve fiber density, although glycemia and weight loss remained characteristic of a severe diabetogenic phenotype. These observations support the hypothesis of neuroprotective mechanisms at least partially independent of glycemic control, probably mediated by direct effects on the neurovascular unit, Schwann cells, and small sensory fibers.

Studies on the antidiabetic effects of chitosan predominantly describe the lowering of blood glucose and the improvement of insulin resistance in models of type 2 diabetes or NIDDM, with the implication of reduced hepatic gluconeogenesis and increased muscle glucose uptake. Studies in diabetic rats have shown that chitosan inhibits PEPCK expression and activates pathways that favor GLUT4 translocation and Akt phosphorylation in skeletal muscle, which translates into improved glucose tolerance [27,33]. In contrast, in our study chitosan did not significantly reduce blood glucose in mice with STZ-induced type 1 diabetes, suggesting that in the context of almost complete β-cell destruction, its metabolic effects are insufficient to normalize blood glucose, but may remain relevant at the tissue level (e.g., in the nerve or liver) by modulating nutrient-dependent signaling pathways and oxidative stress.

The observed hypolipidemic effects—marked reduction in LDL cholesterol, triglycerides and total cholesterol—are, however, consistent with the rich literature highlighting the ability of chitosan to bind bile acids in the intestinal lumen, to reduce lipid absorption and to modify the expression of genes involved in the synthesis and oxidation of fatty acids [34]. Studies in diabetic mice and rats have demonstrated that chronic administration of chitosan or chitosan-based nanoformulations reduces triglycerides and cholesterol, improves the lipoprotein profile, and attenuates hepatic steatosis and renal dysfunction [35,36,37,38,39]. Considering the central role of dyslipidemia in the activation of polyol, AGE, PKC and hexosamine pathways involved in the pathogenesis of diabetic neuropathy, normalization of the lipid profile may reduce lipotoxicity in the vasa nervorum and Schwann cells, diminishing oxidative stress and intraneural inflammation [9].

From a pathophysiological perspective, our data are compatible with the current model of diabetic neuropathy, in which hyperglycemia and dyslipidemia generate oxidative stress, mitochondrial dysfunction, and activation of NF-κB and inflammatory pathways (including TLR4/iNOS) in axons and Schwann cells [40,41,42]. Activation of Nrf2 in peripheral nerve and Schwann cells has been shown to reduce oxidative stress and apoptosis, improving nerve function in models of DPN, while decreased Nrf2 expression is considered a central link in the amplification of inflammation and axonal degeneration [43,44,45]. In this context, the fact that chitosan exerts antioxidant and anti-inflammatory effects by modulating the Nrf2/HO-1 axis and inhibiting NF-κB is particularly relevant for the interpretation of the neuroprotection observed in our study.

Several experimental studies have shown that chitosan and chitooligosaccharides (COSs) reduce the production of NO, PGE_2_ and proinflammatory cytokines (TNF-α, IL-1β, IL-6), increase the expression of HO-1 and antioxidant enzymes (SOD, CAT, GPx), and modulate the Erk1/2, Akt and Nrf2/HO-1 pathways in macrophages and other cell types [46,47,48,49]. Chitosan-based nanoparticles have also been shown to reduce oxidative stress and inflammation in various organs by activating Nrf2 and inhibiting NF-κB, which gives them a pronounced profile of “ROS-scavenging biomaterials” [50, 51]. Although these studies did not directly target the diabetogenic peripheral nerve, the convergence on the Nrf2/NF-κB axis suggests that the same mechanisms may also operate at the level of the sciatic nerve, contributing to the reduction in nitro-oxidation and the protection of myelinated and unmyelinated fibers.

The hypothesis that chitosan acts directly on the peripheral nerve is also supported by the extensive literature on its use as a biomaterial in nerve regeneration. Chitosan is biocompatible with nerve cells and Schwann cells, supports their adhesion and proliferation, and reduces scar formation, and has been used in nerve guides and reconstruction tubes for critical sciatic nerve defects [52,53]. More recently, a systematic review of chitosan-based devices concluded that they improve functional and morphological outcomes of nerve repair, including in experimental diabetic neuropathy [54]. Particularly relevant to the context of diabetes is the fact that repairing nerve defects with chitosan grafts supplemented with vascular stromal fraction improved nerve regeneration in STZ-diabetic rats, suggesting that chitosan can support regeneration even in a metabolically hostile environment [55]. Also, studies using chitosan nerve guides in experimental diabetes models have shown that axonal regeneration and re-myelination can be “equalized” between diabetic and non-diabetic animals when optimized guide configurations are used, suggesting that the microenvironment provided by chitosan partially compensates for the impact of diabetes on Schwann cells [56].

Even though in our study the administration is systemic rather than local, these data support the concept that chitosan interacts favorably with Schwann cells and the extracellular matrix, facilitating the preservation of myelin and axonal integrity, which is reflected in the partial normalization of CMAP amplitude and duration and in the increase in IENF density.

At the molecular level, several lines of evidence indicate that Schwann cells exposed to hyperglycemia undergo oxidative stress and inflammation mediated by TLR4/NF-κB, with reduced Nrf2 activity, which favors apoptosis and myelin loss [43]. Activation of Nrf2 in Schwann cells protects against these changes and improves axonal regeneration. In parallel, recent reviews on the neuroproperties of chitosan highlight that it reduces β-amyloid accumulation, stabilizes neuronal membranes, modulates Ca^2+^ influx and inhibits proapoptotic pathways, showing neuroprotective effects in models of cerebral ischemia, Alzheimer’s disease, and CNS trauma [50].

Applied to diabetic neuropathy, these mechanisms could explain the protection of intraepidermal small fibers observed by us, given that IENF density is a sensitive biomarker of small fiber neuropathy and correlates with pain and the risk of plantar ulcers in diabetes [57,58]. Regarding nociceptive behavior, the pattern of thermal hyperalgesia and mechanical allodynia observed in diabetic mice in our study is congruent with the phenotype described in murine and rat models of painful diabetic neuropathy, in which Von Frey filament and Tail-Flick tests are used to quantify Aδ and C fiber dysfunction [59]. Various interventions with natural compounds—for example naringin, diosgenin, nettle extracts or hesperidin—have demonstrated that the improvement of hyperalgesia and allodynia is closely related to the reduction in oxidative stress and inflammation, through convergent pathways on Nrf2, SIRT1, and NOX4 [60]. The fact that chitosan, when administered orally, produces a similar analgesic profile suggests that it falls into the category of compounds with “disease-modifying” effects on diabetic neuropathy, not just symptomatic, probably through comparable molecular mechanisms [58]. Another aspect worth emphasizing is that the neuroprotection observed in our study is parallel with documented systemic beneficial effects of chitosan in other diabetic complications. Chitosan nanoformulations loaded with selenium or piperine have been shown to improve glycemic control, reduce oxidative stress, and ameliorate cognitive dysfunction in diabetes models, suggesting that the same “package” of antioxidant, anti-inflammatory, and lipid-lowering effects can simultaneously protect the brain, kidney, and peripheral nerves [61]. This multimodality is attractive for diabetic neuropathy, a condition that develops against the backdrop of a profoundly altered systemic “terrain,” in which treatments strictly targeted at the nerve may not be sufficient to stop the pathogenic cascade.

### Limitations of the Study

Despite these arguments, our study has important mechanistic limitations. We did not directly quantify oxidative stress markers, Nrf2 expression, NF-κB, or cytokine levels (such as TNF-α) in sciatic nerve or skin, so attribution of the observed effects to these exact pathways remains inferential, based on analogy with other models and the existing literature for chitosan. We also did not differentiate between possible effects mediated by low- vs. high-molecular-weight fractions or by the degree of deacetylation, factors that strongly influence bioavailability and interactions with cell membranes. We also used a single-sex, a single-diabetes model (STZ, type 1), and a single dose of chitosan; it remains to be determined whether the same magnitude of effects is found in female type 2 diabetes models or at other dosage levels and formulations (e.g., nanochitosan, COS). Future studies should also compare chitosan with established neuroprotective agents such as alpha-lipoic acid and duloxetine, while evaluating the long-term safety and tolerability of chronic oral administration. STZ induces near-complete β-cell destruction and mimics autoimmune-like T1DM rather than insulin-resistant T2DM. Although DPN shares common mechanisms across diabetes types (oxidative stress, inflammation, dyslipidemia), metabolic context differs. For translational relevance, T2DM models such as high-fat diet + low-dose STZ or db/db mice may yield complementary information. Finally, we acknowledge that CMAP amplitude and duration, while useful indicators of peripheral nerve function, may also be influenced by neuromuscular or muscle-level factors; therefore, the absence of nerve conduction velocity (NCV) measurements represents a methodological limitation of this study, which we aim to address in future experiments by integrating NCV alongside CMAP analysis.

Looking ahead, future studies should integrate detailed molecular analyses at the peripheral nerve level (cytokine profile, Nrf2/HO-1 markers, iNOS, AGEs/RAGE, neurotrophic factor expression) and in vitro experiments on Schwann cells exposed to hyperglycemic conditions in the presence or absence of chitosan, to clarify whether the dominant effects are antioxidant, anti-inflammatory, pro-regenerative, or a combination thereof. In addition, longitudinal correlation of IENF density with electrophysiological and behavioral parameters, inspired by clinical studies using skin biopsy as the gold standard for small fiber neuropathy, could provide a more complete picture of how chitosan modulates neuropathy progression.

Clarifying these aspects will be essential to transform chitosan from a promising biomaterial into a realistic candidate for disease-modifying therapies in diabetic peripheral neuropathy.

## 5. Conclusions

The present study demonstrates that long-term oral administration of chitosan exerts significant neuroprotective effects in a murine model of STZ-induced diabetic neuropathy, despite the maintenance of a severe hyperglycemic profile. Chitosan ameliorated sensory dysfunction (thermal hyperalgesia and mechanical allodynia), improved mobility and exploratory behavior, partially restored sciatic nerve electrophysiological parameters, and preserved intraepidermal nerve fiber density. In parallel, the treatment markedly reduced diabetes-associated dyslipidemia, normalizing LDL cholesterol, triglycerides, and total cholesterol levels, suggesting a beneficial systemic impact on the metabolic milieu that influences neuropathy progression. Taken together, these results indicate that chitosan acts through mechanisms that are at least partially independent of glycemic regulation and can modulate both the functional and structural components of diabetic neuropathy. Given its antioxidant, anti-inflammatory, hypolipidemic, and nerve regeneration-compatible properties, chitosan emerges as a potential disease-modifying agent in diabetic neuropathy. However, further studies are needed to elucidate the molecular mechanisms involved, assess dose dependence, test in type 2 diabetes models, and validate the results with specific biomarkers of oxidative stress and inflammation.

## Data Availability

The original contributions presented in this study are included in the article. Further inquiries can be directed to the corresponding author.
